# *Escherichia coli* Nissle 1917 Occupies Previously Undocumented Host Niches in the Insect‐Parasitic Nematode *Steinernema hermaphroditum*

**DOI:** 10.1111/1758-2229.70326

**Published:** 2026-04-05

**Authors:** Victoria Chen, John P. Marken, Richard M. Murray, Mengyi Cao

**Affiliations:** ^1^ Department of Biological Engineering Massachusetts Institute of Technology Cambridge Massachusetts USA; ^2^ Section of Microbiology University of Copenhagen Copenhagen Denmark; ^3^ Division of Biology and Biological Engineering California Institute of Technology Pasadena California USA; ^4^ Division of Biosphere Sciences and Engineering Carnegie Institution for Science Pasadena California USA

**Keywords:** coelomocyte, cuticle, entomopathogenic, *Escherichia coli* Nissle 1917, intestine, nematode, *Steinernema hermaphroditum*

## Abstract

*Steinernema* species are soil‐dwelling, insect‐parasitic nematodes that maintain species‐specific associations with *Xenorhabdus* symbiotic bacteria, which are packaged within anterior intestinal pockets during the infective juvenile (IJ) stage. While these nematodes can persist in soil for months while seeking insect hosts, their interactions with environmental microbes beyond their native symbionts remain poorly understood. Here, we describe a previously uncharacterized interaction between *Escherichia coli* Nissle 1917 (EcN) and *Steinernema hermaphroditum*. EcN cells are enclosed and lysed within multiple pairs of putative coelomocytes, suggesting microbial endocytosis by host cells. During the IJ stage, EcN localizes to posterior intestinal compartments and the inter‐cuticular space, where cells proliferate, aggregate and subsequently lyse. Bacterially expressed proteins persist within the nematode cuticle for over 8 weeks in non‐sterile soil. These findings reveal sequential stages of environmental bacterial colonization associated with host immune responses distinct from mutualistic symbiosis. This work establishes a model for understanding nematode and environmental microbe interactions and highlights opportunities to deliver bacterially expressed molecules for environmental biosensing and biocontrol applications.

## Graphical abstract

This study characterizes previously undocumented host tissues in *Steinernema* nematodes, including putative coelomocytes, intestinal compartments, and inter‐cuticular spaces colonized by *Escherichia coli* Nissle 1917. Our findings reveal niche‐specific microbial interactions distinct from those with native symbionts, establishing a platform for environmental microbial engineering using *Steinernema* nematodes.

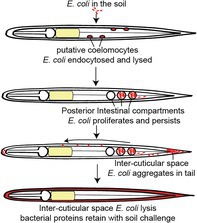

## 1 Introduction

Animal tissues create complex, compartmentalized spaces that provide abiotic and biotic environments capable of selecting and maintaining mutualistic microorganisms while antagonizing pathogenic ones. Reciprocally, microbes develop within these host‐derived environments, shaping the identity of the niches they occupy (Baquero et al. 2021). Identifying and characterizing novel niches within animal hosts, as well as the dynamic interactions of microbes within these microenvironments, are crucial for understanding the mechanisms and consequences of host‐microbe crosstalk. Binary symbioses, involving a single core symbiont and a single animal species, provide efficient models for revealing the temporal and spatial dynamics of symbiont colonization within specific host cells and tissues. The niche‐specific interactions observed in diverse binary symbiosis models are critical for host development and ecosystem health (Maire et al. 2020; Nyholm and McFall‐Ngai 1998; Stilwell et al. 2018; Tivey et al. 2022). While binary symbiosis often involves colonization by a single microbial species within a specialized organ, additional genera or species can sometimes associate with distinct tissues, maintaining spatial separation from primary symbionts (Dale and Moran 2006; Nyholm and McFall‐Ngai 2021). The relatively simple composition of these natural microbiomes makes such animal models advantageous for studying multiple types of host–microbe interactions simultaneously.

**FIGURE 1:**
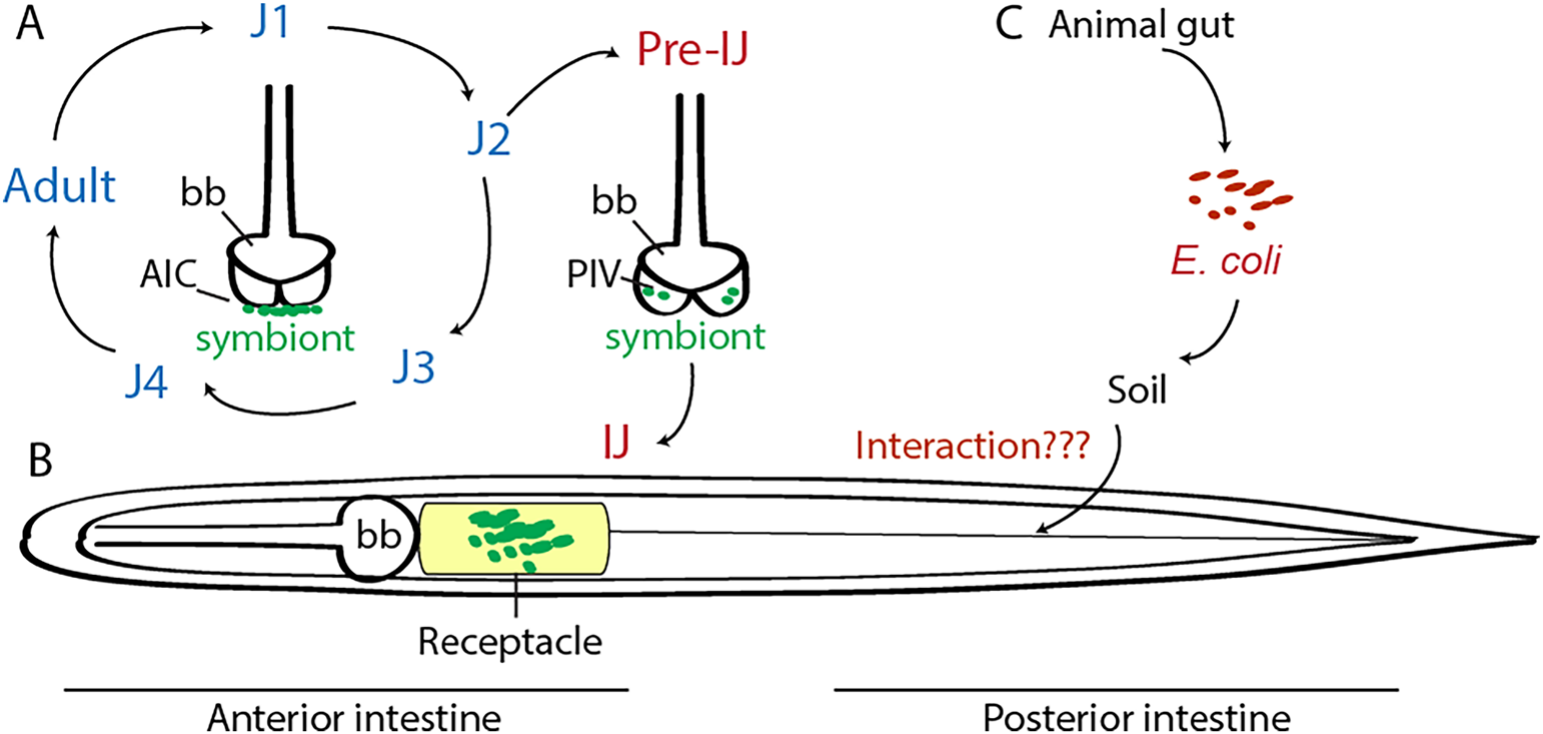
*Steinernema hermaphroditum* intestine provides specific niches for host–microbe interactions. (A) A simplified diagram of *S. hermaphroditum* life cycle and symbiont colonization in specific tissues at the anterior intestine. AIC: anterior intestinal caecum; PIV: pharyngeal intestinal valve. (B) *S. hermaphroditum* IJ that has the potential to interact with both mutualistic *Xenorhabdus* bacteria and other microbes. Bb: basal. (C) Biphasic lifestyle of *E. coli* that oscillates between associating with mammalian gut and persisting in the soil, which provides an opportunity to interact with soil‐dwelling *Steinernema* nematodes.

The entomopathogenic (EPN, insect‐parasitic) nematode *Steinernema* spp. maintains a stable association with mutualistic *Xenorhabdus* bacteria in a binary symbiosis. Each generation of nematodes acquires their symbiotic bacteria from the external environment (semi‐horizontal) while a maternal factor can influence the bacterial colonization of the next generation (semi‐vertical). The intestine of *Steinernema* nematodes contains compartmentalized niches where multiple microenvironments enable the attachment, persistence, and proliferation of *Xenorhabdus* bacteria (Chaston et al. 2013; Figure 1A,B). *Xenorhabdus* bacteria first attach to the anterior intestinal caecum (AIC) in juveniles, while nematodes feed on bacteria, moult through four juvenile stages (J1–J4) and become adults to reproduce. Signals from overcrowding and lack of food source induce the J2 stage of juveniles to moult into infective juvenile (IJ)s through an alternative developmental pathway. During IJ development, the symbiotic bacteria first localize to the pharyngeal intestinal valves (PIV) in the pre‐IJs. These narrow pouches restrict bacterial colonization to a few cells per animal. During the IJ stage, symbiotic bacteria migrate into the anterior intestinal pocket, termed the receptacle (Figure 1B). The multiple intestinal tissues (niches) involved in the colonization events, including AIC, PIV and the receptacle, are proposed to provide specific nutrients, signalling molecules, and physical restrictions to select the specific species or strain of *Xenorhabdus* bacteria.

Our knowledge of niche‐specific colonization in *Steinernema* is limited to its interaction with *Xenorhabdus*. However, *Steinernema*, as soil‐dwelling nematodes, also encounter other species of microbes from their natural environment. Bacteria from multiple genera, termed the ‘pathobiome’, are found to frequently associate with certain *Steinernema* spp. and potentially contribute to entomopathogenic (insect‐killing) activities (Ogier et al. 2020). Despite its generalization of pathobiomes among multiple *Steinernema* species, the frequency, localization and molecular mechanisms of pathobiome association are unknown. Little else is known about other environmental microbes, either pathogenic, mutualistic or commensal, that interact with EPNs or if the host nematode could possibly create other niches beyond AIC, PIV or IJ receptacle for such interactions. This knowledge gap limits the full application of *Steinernema* as a symbiosis model and its potential applications in bioengineering. Therefore, understanding the spatial and temporal interactions of pathogens and commensals in *Steinernema* provides a foundational framework for novel modes of symbiotic interactions and will expand our knowledge of host niches beyond mutualistic interaction.

*Escherichia coli* is the most established bacterial species for molecular biology and is a leading candidate for bioengineering applications across diverse niches, including biosensing in complex microenvironments (Valle et al. 2021) and modifying gene expression in multicellular organisms (Gao and Sun 2021). *E. coli* is commonly associated with the animal intestinal tract (host‐dependent) and circulates through the soil and water environment (host‐independent) via a biphasic lifestyle (Figure 1C). Certain *E. coli* strains can persist in soil environments, where they influence indigenous microflora encountered by insects and soil‐dwelling nematodes (van Elsas et al. 2010). The *E. coli* Nissle 1917 (EcN) strain, originally isolated from a soldier who survived a *Shigella* endemic, is known to interact with the mammalian intestinal epithelium and mucosal immune system in a non‐pathogenic manner, antagonize pathogenic enterobacteria, and confer probiotic benefits in humans (Sonnenborn 2016). Engineered EcN have been used in mammalian systems to combat pathogens (Hwang et al. 2017), detect biomarkers for cancer (Danino et al. 2015) and deliver therapeutic payloads (Harimoto et al. 2022) and have even been used in human critical trials to treat metabolic diseases (Kurtz et al. 2019; Vockley et al. 2023).

In adults of the model nematode *Caenorhabditis elegans* , EcN as a food source was observed to show both probiotic and neurodegenerative effects (Kim and Moon 2019; Redweik and Xue 2025). Engineered EcN has also been used as biosensors to monitor the *C. elegans* intestinal environment (Rutter et al. 2019). Like *C. elegans* , *Steinernema hermaphroditum* also exhibits the properties of transparency, a short in vitro life cycle and a bacteriovorous nature that make it an ideal system for interacting with engineered EcN. Furthermore, because of *Steinernema*'s established usage as a biocontrol agent in agriculture (Shapiro‐Ilan et al. 2025), understanding the interactions between EcN and *Steinernema* spp. would be particularly useful for establishing a foundation for potential synthetic biology solutions to challenges in sustainable agriculture (Jones et al. 2023). However, the interactions of EcN with nematode species beyond *C. elegans* are still underexplored.

In this study, we investigated the interactions between EcN and the entomopathogenic nematode *Steinernema hermaphroditum*. We discovered that EcN localizes within previously undocumented cell and tissue types in *S. hermaphroditum*, including putative immune cells (coelomocytes), posterior intestinal tissues, and inter‐cuticular spaces. These snapshots of EcN localization in nematodes suggest a sequential process of cell‐ and tissue‐specific microbe‐host interactions during IJ development. Notably, EcN colonization occurs in spatially distinct tissues from those occupied by *Xenorhabdus*, the nematode's native symbiont. Here we show *E. coli* Nissle‐*S. hermaphroditum* interactions as niche‐specific, a potential model for novel host–microbe interactions that can be applied in environmental engineering.

## 2 Experimental Procedures

### 2.1 Construction of Fluorescent *E. coli* Nissle Strains With mScarlet‐I

Genetic constructs for constitutive expression of red fluorescent protein (mScarlet‐I) was assembled using the 3G assembly method (Halleran et al. 2018) with standard parts from the MoClo modular cloning toolkit (Iverson et al. 2016). The resulting constructs were cloned into the ‘KL’ genome integration vector from the pOSIP one‐step genome integration system (St‐Pierre et al. 2013), which facilitates integration at the lambda integrase attachment site in the *E. coli* genome. Plasmids were transformed into chemically competent *E. coli* strains (Nissle 1917) CSH50 (Sokurenko et al. 1992), and BW25113 (Grenier et al. 2014) using a standard heat shock protocol. Briefly, 50 μL of competent cells were mixed with 100 ng of plasmid DNA, incubated on ice for 30 min, heat shocked at 42°C for 45 s, and then placed back on ice for 2 min. Cells were allowed to recover in 950 μL of SOC medium at 37°C with shaking for 1 h before being plated on LB agar plates containing kanamycin (50 μg/mL) to select for successful integrants. Plates were incubated at 37°C overnight. Resulting transformants were screened via colony PCR on the integration locus using the standard screening primers from (St‐Pierre et al. 2013) and Sanger sequenced to validate the successful integration.

### 2.2 Liver‐Kidney Agar Plates

The recipe for Liver‐kidney agar was adapted from (Flores‐Lara et al. 2007): per Litre of autoclaved media contains 50 g of homogenized pork liver and 50 g of homogenized pork kidney, 2.5 g NaCl, 7.5 g agar and water. To avoid contamination, the liver‐kidney agar was supplemented with kanamycin (50 μg/mL) and handled under a sterile fume hood.

### 2.3 *S. hermaphroditum* Axenic Egg Extraction and Colonization Assay

Conventional *S. hermaphroditum* nematodes were maintained by propagating through *Galleria mellonela* 5th instar larvae (Cao et al. 2021). The method for axenic egg extraction for *S. hermaphroditum* was adapted from. Liquid cultures of symbiotic bacterium *X. griffiniae* (HGB2511) were grown overnight in LB media that were kept in the dark and added to lipid agar plates (Vivas and Goodrich‐Blair 2001; Cao et al. 2021). Plates were cultured for 48 h to form a bacterial lawn. To prepare axenic (germ‐free) eggs, conventional IJs (exclusively propagated through insects) were added to the lawns of *X. griffiniae* (HGB2511) and incubated for 2–3 days to grow to gravid adults. Nematodes were washed off the bacterial lawn with water and then transferred to 50 mL conical tubes (Falcon, Corning, NY) and centrifuged at 3000 rpm for 2 min. The supernatant was removed using a vacuum, and 2 mL of a lysis solution (2.4 mL 8% bleach, 500 μL 5 M KOH, 7.1 mL water) was added to lyse the gravid adult hermaphrodites. Nematodes were incubated in the lysis solution for 5 min at room temperature and mixed several times per minute by inverting. The solution was then centrifuged at 3000 rpm for 2 min, and the lysis solution was removed using a vacuum. Nematode eggs were washed thrice with 10 mL of LB medium. Their density was assessed by viewing the number of IJs in 2 μL spots under a stereoscope. Axenic eggs were incubated on LB agar overnight at 30°C to confirm there was no contamination.

For colonization assays, approximately 500 eggs were added to the prepared liver‐kidney or lipid agar plates, with or without a bacterial lawn of *X. griffiniae* expressing GFP (Thomas et al. 2024) or *E. coli* expressing mScarlet‐I and incubated at 25°C for 1 week. Plates were then moved to 100 × 25 mm Petri dishes filled with 10–15 mL of water to trap IJs. Three biological replicates of each colonization condition were screened to identify colonization phenotypes based on the presence of EcN in different tissues. Colonization phenotypes and frequencies were assessed using fluorescence microscopy (See Microscopy and Image Acquisition for details).

### 2.4 Calculation of CFU Per IJ

The CFU per IJ assessment was adapted from (Murfin et al. 2019). IJ suspension from water‐trap was added to sterile 1.5 mL microfuge tubes (Eppendorf, Germany) and centrifuged at 634 g for 2 min to concentrate the nematodes. The supernatant was removed using a vacuum, and 1 mL of a 1% bleach solution was added to the pellet, which was incubated for 2 min at room temperature to surface sterilize IJs. IJs were washed thrice with and resuspended in 1 mL of LB medium, and their concentration was assessed by viewing 2 μL spots under a stereoscope. 200 IJs were then resuspended in 250 μL of LB broth. IJs were homogenized for 2 min using a hand‐held motor‐driven grinder and Kontes polypropylene microtube pellet pestle. Serial dilutions of the homogenate were then plated on LB agar plates with 50 μg/mL kanamycin. This surface sterilization protocol was used to confirm live EcN cells could be recovered from intestinally colonized IJs. Since bleaching removes IJ outer cuticle and cuticular associated bacteria, we assessed EcN colonization in the IJ cuticle based on fluorescence microscopy exclusively, not by CFU counting.

### 2.5 Soil Challenge of IJ Colonization

IJ suspensions were transferred to sterile 15 mL conical tubes and centrifuged at 3000 rpm for 2 min to form a pellet. IJs were then resuspended in 10 mL of M9 buffer (Stiernagle 2006) and split into two 50 mL culture flasks, 5 mL each. The concentrations of IJs in each flask were assessed under a stereoscope and recorded. For each plate, a 1:5 dilution of M9‐soaked soil (5 g Miracle‐Gro Potting Mix in 45 mL of M9 incubated at room temperature overnight, approximately 8000 CFU/μL) was filtered through a Whatman paper and added to one of the flasks to simulate microbial challenge. Weekly, the colonization frequency for each flask was measured via fluorescence microscopy by counting the number of colonized and uncolonized worms. 200 IJs were removed from the flask, paralyzed with 2 mM levamisole in 10 μL of sterile water, and imaged on 5% agar pads.

### 2.6 Microscopy and Image Acquisition

IJ nematodes were concentrated from water‐traps by centrifugation at 1761 g for 1 min and immobilized by addition of levamisole to a final concentration of 5 mM. The nematodes were mounted onto 5% agarose in water and covered with a cover glass (Cao et al. 2021). Photomicrographs and videos were acquired using Zeiss Imager Z2 microscope equipped with Apotome 2 and Axiocam 506 mono using Zen 2 Blue software.

## 3 Results

### 3.1 *E. coli* Nissle Cells Interact With Putative Coelomocytes of *S. hermaphroditum* IJ

**FIGURE 2:**
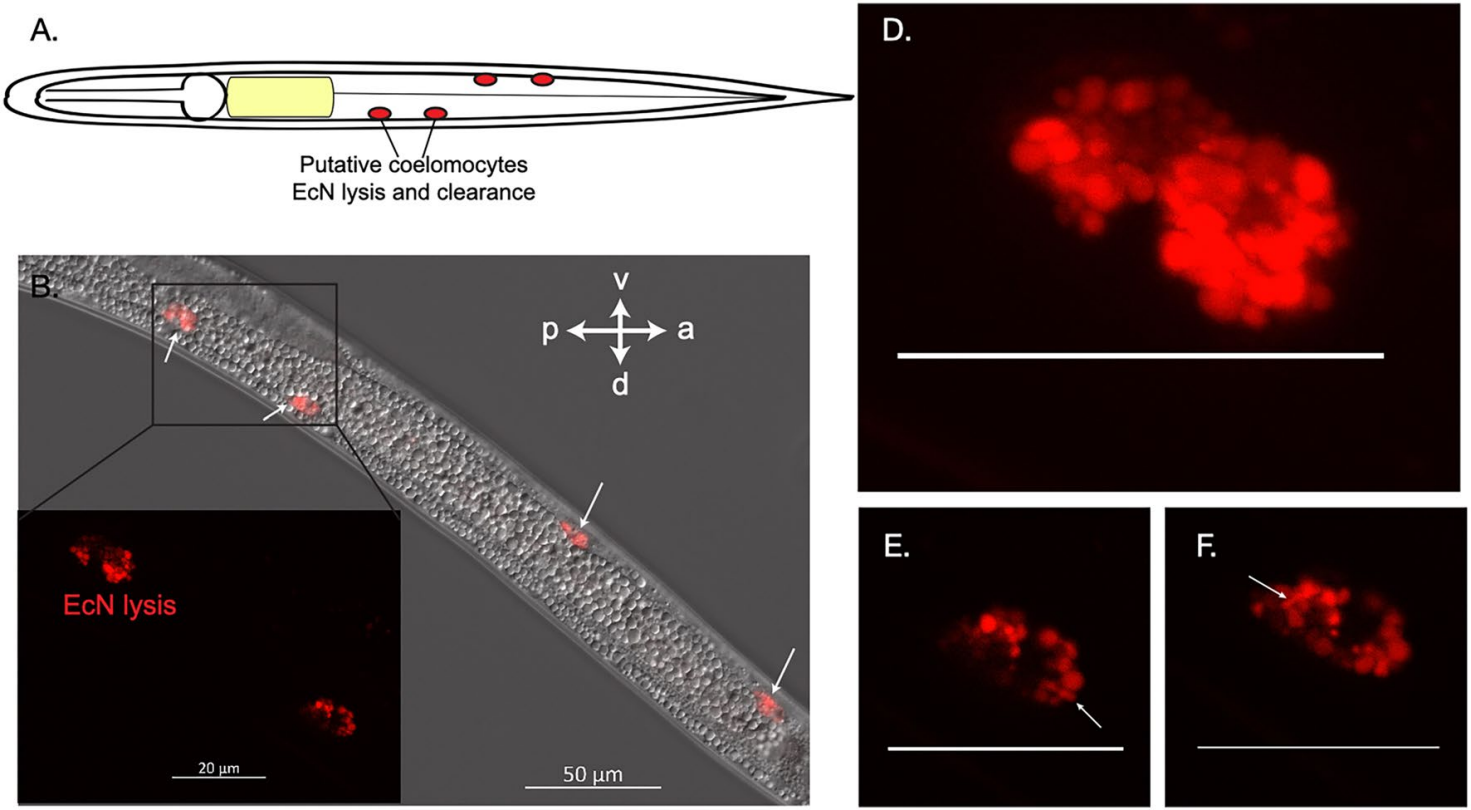
*E. coli* Nissle 1917 (EcN) localizes in the *Steinernema hermaphroditum* IJ putative coelomocytes. (A) A schematic diagram showing *S. hermaphroditum* IJs has at least four pairs of this cell type. (B, C) A representative IJ showing *E. coli* Nissle localized and lysed within putative coelomocytes. (D) Each structure contains two cells. (E, F) Rod‐shaped bacterial cells are observed inside of the host cell cytosol. Scale bars in panel D–F equals to 20 μm. (Also see Video S1).

Using fluorescence microscopy, we observed that mScarlet‐I‐expressing EcN cells localize to at least four ‘spots’ on the lateral sides of *S. hermaphroditum* IJs, with two spots on the posterior dorsal side and two on the anterior ventral side (approximately 5–10 μm in diameter) (Figure 2A,B). Z‐stack imaging for these four ‘spots’ showed that each consists of a pair of large ovoid cells (Figure 2D; Video S1). Within each cell, the cytosol, containing lysates from fluorescent bacteria, surrounds a dark centre which features the nuclease (Figure 2D). Based on the morphology and localization of these cells, we hypothesize that they are coelomocytes, a cell type known to serve defence and immune functions in invertebrates including some nematode species (Engelmann et al. 2005; Tahseen 2009; Smith et al. 2010). Nematode coelomocytes are known to have diverse morphologies, sizes, and numbers. The morphology of coelomocytes ranges from oval‐shaped to star‐shaped; the sizes of these cells correlate with the size of the animals (from less than 1 mm to a few metres long). They are proposed to participate in the uptake and scavenging of proteins circulating in the pseudocoelomic space. Whether or not the coelomocytes could engulf bacterial cells depends on the species of nematode (Tahseen 2009). For instance, *C. elegans* coelomocytes were found to only have endocytosed toxins, such as nematode‐expressed green fluorescent proteins that circulate in the body cavity, but not live microbial pathogens (Fares and Greenwald 2001). In contrast, the mammalian parasitic nematode *Ascaris suum* was observed to phagocytose living bacterial cells in their coelomocytes (Tahseen 2009). Neither the morphology nor the function of *Steinernema* coelomocytes has been previously documented. Consistent with our hypothesis, we observed that the putative *S. hermaphroditum* coelomocytes are oval‐shaped, like other *Rhabditis* nematodes; rod‐shaped bacterial cells were visible in the putative coelomocytes (Figure 2C,E,F), possibly because bacteria are trapped and lysed in the lysosomes. These observations suggest the phagocytotic and endocytic functions of putative *Steinernema* coelomocytes directly towards EcN which may cause an active immune response in the host nematode.

### 3.2 *E. coli* Nissle Cells Localize and Proliferate in the IJ Intestine

**FIGURE 3:**
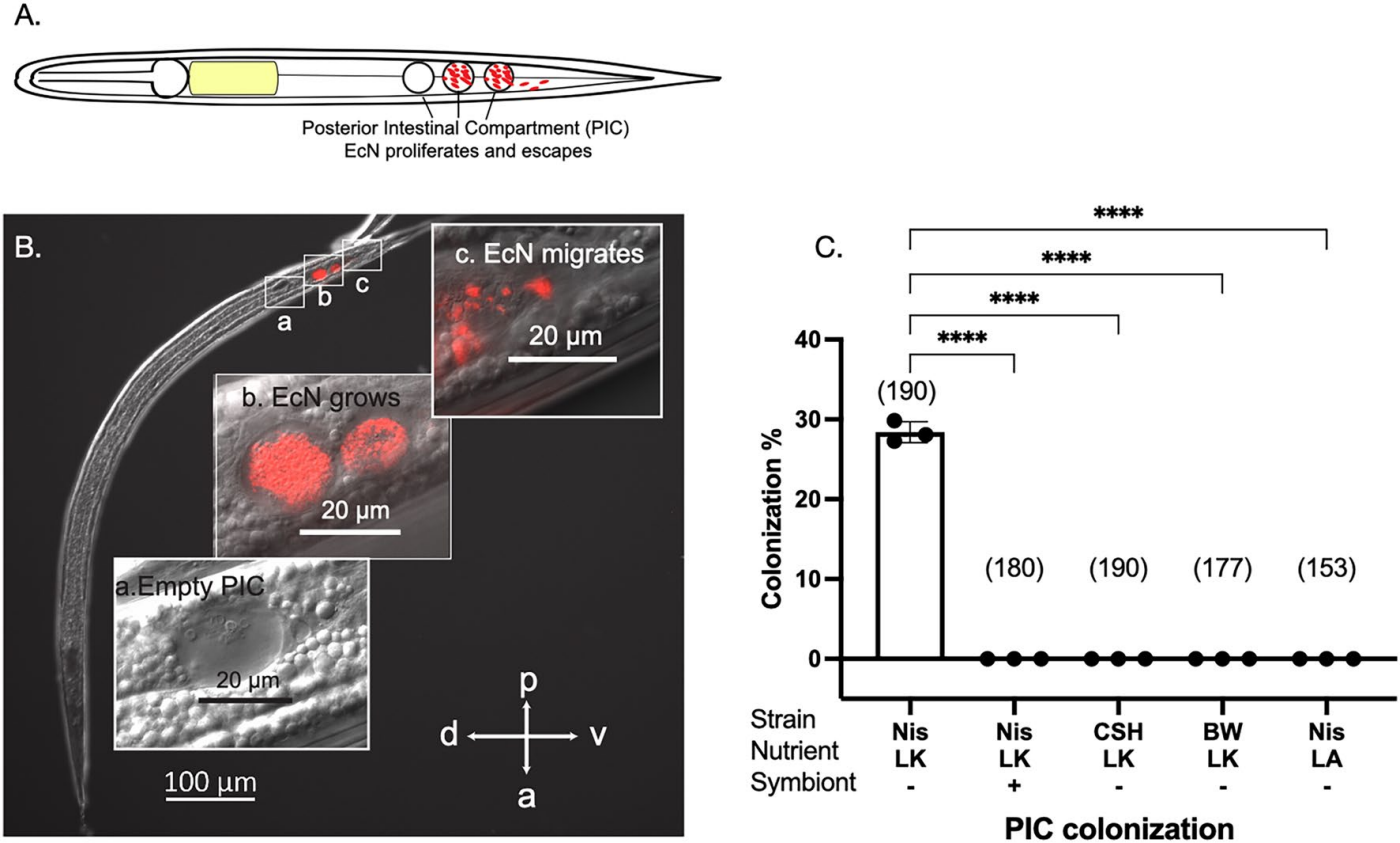
*E. coli* Nissle localizes in the *S. hermaphroditum* intestinal lumen and posterior intestinal compartments. (A) A schematic diagram showing *E. coli* Nissle colonizing nematode intestine and proliferating in the posterior intestinal compartments. (B) A representative IJ showing intestinal colonization of bacteria: an empty intestinal compartment containing cell or organelle debris‐looking substance (a); bacterial colonization in the IJ posterior intestinal compartments (b); bacterial cells escape from the posterior intestinal opening (c). (C) Quantification of intestinal colonization frequency of *E. coli* strains: Nissle (Nis), *E. coli* CSH50 (CSH), BW25113 (BW) under nutritional conditions of either liver‐kidney (LK) or lipid agar (LA). Treatments were either with (+) or without (−) symbiont *X. griffiniae*. Numbers in the parenthesis indicate the sample size (*n* = number of animals screened in each treatment). Each data point represents one biological replicate (a batch of nematodes) from one independent experiment. Three biological replicates are shown with mean and standard deviation. Statistical analysis was performed by one‐way ANOVA. *p* < 0.0001 (****).

As typical of bacterivorous nematodes, the intestine of *Steinernema* serves as the largest organ facilitating microbial interactions where bacteria can be engulfed, digested or colonized. Prior to IJ development, EcN cells are engulfed and digested in the intestinal lumen of *S. hermaphroditum* juveniles (Figure S2). When the intestinal lumen collapses and closes during IJ development, we observe EcN cells attaching to the posterior end of the IJ intestine within multiple small ‘pockets’ that are not identified previously. We term these structures ‘Posterior Intestinal Compartments’, ‘PIC’ (Figure 3A,B). Within the same IJ, EcN cells localize and proliferate in one or more compartments (Figure 3B), showing their role as potential reservoirs for microbes. Individual bacterial cells were also observed to ‘escape’ from these compartments at the posterior end, pass through the anus, and eventually enter the space in between the internal cuticle and a layer of growing exterior cuticle (Figure 3B,C). This process may be caused by either bacterial motility (such as swimming) or by the movement of the nematode intestine during IJ development as it collapses and squeezes out the bacteria accumulated inside the intestinal lumen. Intestinal colonization was not observed for two other tested laboratory strains of *E. coli* : strain BW25113 and strain CSH50, suggesting that EcN contains genetic factors that allow colonization not found in these other laboratory strains (Figure 3C). Intestinal colonization was specific to liver‐kidney agar condition lacking *X. griffiniae* symbiotic bacteria (Figure 3C). Liver‐Kidney agar is a complex, undefined medium that may provide specific nutrients that are not available from lipid agar media. Such nutritional effect may alter nematode development or physiology or modulate EcN physiology such as the production of virulence factors. The addition of the *X. griffiniae* symbiont was also shown to inhibit intestinal colonization of EcN (Figure 3C), possibly due to its ability to antagonize *E. coli* growth by producing antimicrobial molecules. Overall, our data suggest that EcN can actively breach niches within *S. hermaphroditum*, thereby prompting a host immune response that can nonetheless be evaded by some EcN cells that persist in the IJ intestine.

### 3.3 *E. coli* Nissle Cells Aggregate and Migrate in the IJ Inter‐Cuticular Space

**FIGURE 4:**
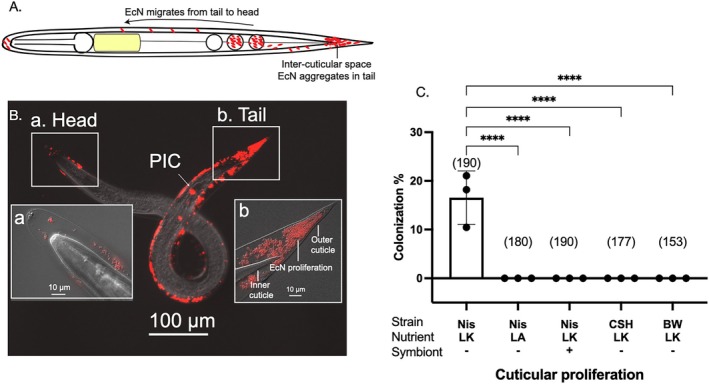
Intercuticular colonization of EcN. (A) A schematic diagram showing *E. coli* Nissle colonizes in the intercuticular space in the IJs. (B) A representative IJ showing intercuticular proliferation of bacteria: the head region (a) and the tail region (b). Arrow indicates co‐occurrence of colonization at the posterior intestinal compartments and intercuticular space in the same IJ. (C) Quantification of cuticular proliferation frequency of *E. coli* strains: Nissle (Nis), *E. coli* CSH50 (CSH), BW25113 (BW) under nutritional conditions of either liver‐kidney (LK) or lipid agar (LA). Treatments were either with (+) or without (−) symbiont *X. griffiniae*. Numbers in the parenthesis indicate the sample size (*n* = number of animals screened in each treatment). Each data point represents one biological replicate (a batch of nematodes) from one independent experiment. Three biological replicates are shown with mean and standard deviation. Statistical analysis was performed by one‐way ANOVA. *p* < 0.0001 (****).

*S. hermaphroditum* IJs can have up to two cuticle layers: an inner collagenous cuticle similar to that seen in other developmental stages (Wolkow and Hall 2011), and sometimes an outer cuticle that is known to protect the animal from pathogenic microbes (Timper and Kaya 1989). We observed aggregates of bacterial cells trapped in the posterior end of the inter‐cuticular space (Figures 4A,B and S3). Cuticular association of EcN is either exclusively localized to the posterior side or co‐localized at both the posterior and anterior sides (Figure S3). No IJ was only associated at the anterior cuticle without posterior colonization, confirming the directionality of colonization from the posterior to the anterior; thus, this event must follow intestinal colonization. Like colonization of the posterior intestinal compartments, inter‐cuticular colonization was also specific to growth on liver‐kidney agar without the presence of any *X. griffiniae* symbionts, and was not observed for the laboratory *E. coli* strains BW25113 or CSH50 (Figure 4C). This is consistent with our hypothesis that these two colonization phenotypes are correlated (Figures 3C and 4C). Along this line of evidence, we also observed the co‐occurrence of EcN cells during intestinal and cuticular colonization (Figure S3).

### 3.4 Proteins From *E. coli* Cell Lysates Persist in the IJ Inter‐Cuticular Space

**FIGURE 5:**
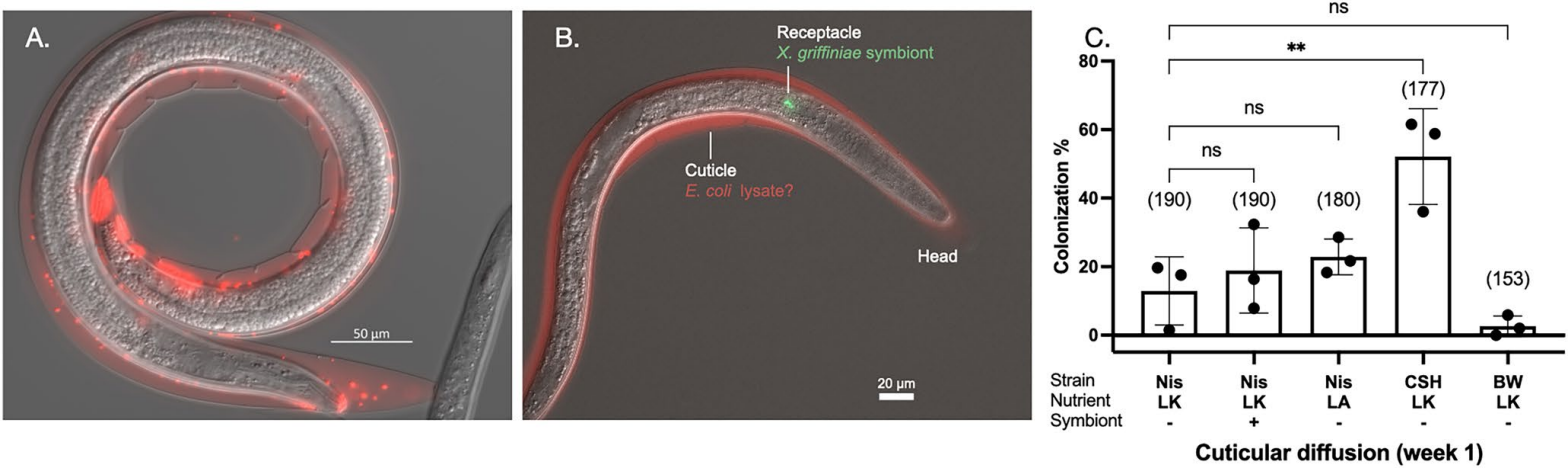
*E. coli* Nissle produced mScarlet‐I proteins persist in the IJ inter‐cuticular space. (A) A representative IJ (2 weeks post trapping) showing transitional stage in which bacterial cells and mScarlet‐I contained in cell lysate co‐localize in the inter‐cuticular space. (B) Diffused fluorescence from mScarlet‐I produced by *E. coli* cells is found in symbiont‐colonized IJs. (C) Quantification of cuticular diffusion of mScarlet‐I expressing *E. coli* strains: Nissle (Nis), *E. coli* CSH50 (CSH), BW25113 (BW) under nutritional conditions of either liver‐kidney (LK) or lipid agar (LA). Treatments were either with (+) or without (−) symbiont *X. griffiniae*. Numbers in the parenthesis indicate the sample size, *n* = number of animals screened in each treatment. Each data point represents one biological replicate (a batch of nematodes) from one independent experiment. Three biological replicates are shown with mean and standard deviation. Statistical analysis was performed by one‐way ANOVA. *p* < 0.01 (**).

We observed live EcN cells that were temporarily maintained in the IJ cuticular space for approximately 2 weeks before they were observed to lyse. Lysis is marked by the presence of both individual bacterial cells and a smear of bright fluorescence from the mScarlet‐I reporter (Figure 5A). Eventually, no individual living mScarlet‐I‐tagged bacteria are observed in the intercuticular space, suggesting all the observed fluorescence comes from residual mScarlet‐I proteins persisting in the bacterial lysate (Figure 5B). Surprisingly, diffused mScarlet‐I fluorescence is observed in the inter‐cuticular space in IJs grown under all conditions (Figure 5C), including those where cuticular proliferation of living bacteria was not observed (Figure 4C). This observation suggests that in addition to living bacterial colonization as described above, other mechanisms could cause bacterial proteins to be expelled and trapped in the inter‐cuticular space.

**FIGURE 6:**
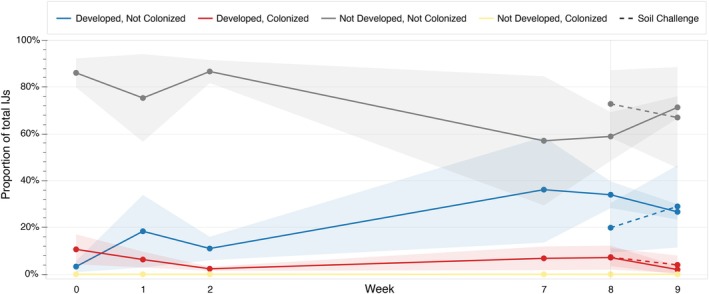
Proportion of developed outer cuticles and cuticularly colonized IJs as a fraction of total IJs with and without soil challenge over 9 weeks. Developed cuticles (noted as ‘developed’) include those with fully developed, less developed, or broken outer cuticles. The presence of *E. coli* Nissle produced mScarlet‐I proteins in IJ cuticles (noted as ‘colonized’) was assessed by fluorescence microscopy. Data are plotted as the mean (lines and points) ± SEM (shaded area) from three biological replicates. A comparison of IJ cuticle development and cuticular colonization by mScarlet‐I in weeks 8 and 9 following challenge with non‐sterile soil is shown as dashed lines.

We investigated if fluorescent proteins contained in the nematode cuticle can persist long‐term when the IJs were challenged by external soil microbes (Figures 6 and S4). Although cuticle growth and colonization percentages varied among different replicates and experiments within populations of IJs, mScarlet‐I proteins in the lysates remained fluorescent in the cuticle over 8 weeks of IJ ageing (Figures 6 and S4). Overall, soil challenge did not significantly affect mScarlet‐I persistence in the cuticle.

## 4 Discussion

In this research, we found EcN cells interacting with four pairs of putative coelomocytes in *S. hermaphroditum*. In the model organism *C. elegans* that has the most characterized anatomy and immune system among nematode species, three pairs of coelomocytes are attached to the pseudocoelomic space with three layers of defence preventing bacterial access: the grinding of bacteria by the pharynx, multiple layers of cuticles and an active immune system defending against the invading microbes (Altun and Hall 2005). Here, we observed that EcN can access structures that we propose to be *S. hermaphroditum* coelomocytes based on the cell morphology and the phagocytic function. However, based on the cell location, they could also be possible intestinal cells. The identity of this cell type needs to be confirmed by future work, such as phagocytosis assays and transcriptional markers. If these cells are truly coelomocytes and serve an immune function, our observation suggests that at least one of the three barriers fails to defend against EcN. Invertebrate immune cells are known to interact with both mutualistic and pathogenic microbes. For instance, in the Hawaiian bobtail squid *Euprymna scolopes* , symbiotic *Vibrio fischeri* bacteria adhere to a single blood‐cell type, the haemocytes, with less affinity in comparison to pathogen adherence (Nyholm et al. 2009). In *S. hermaphroditum*, the mutualistic symbiont *X. griffiniae* has not been reported or observed to interact with the cell type that we propose to be host coelomocytes, suggesting these cells may not respond to native symbiotic bacteria in the same manner as EcN.

EcN cells were also found enclosed in the posterior intestinal pockets of IJs. These pockets, which we term ‘Posterior Intestinal Compartments’, or ‘PIC’ are shown in conventional IJs (those caught from the wild and grown only through the natural insect infection process), suggesting that these structures may be generalized in the host animals in nature. The presence of debris‐like material suggests these structures may form through a process involving cell or organelle degradation (Figures 3A,B and S1; Videos S2 and S3), but their origin remains to be determined. Our data also suggest that the posterior end, similar to the anterior end of the IJ intestine (Chaston et al. 2013), features compartments that are specific to the colonization and proliferation of certain environmental microbes.

**FIGURE 7:**
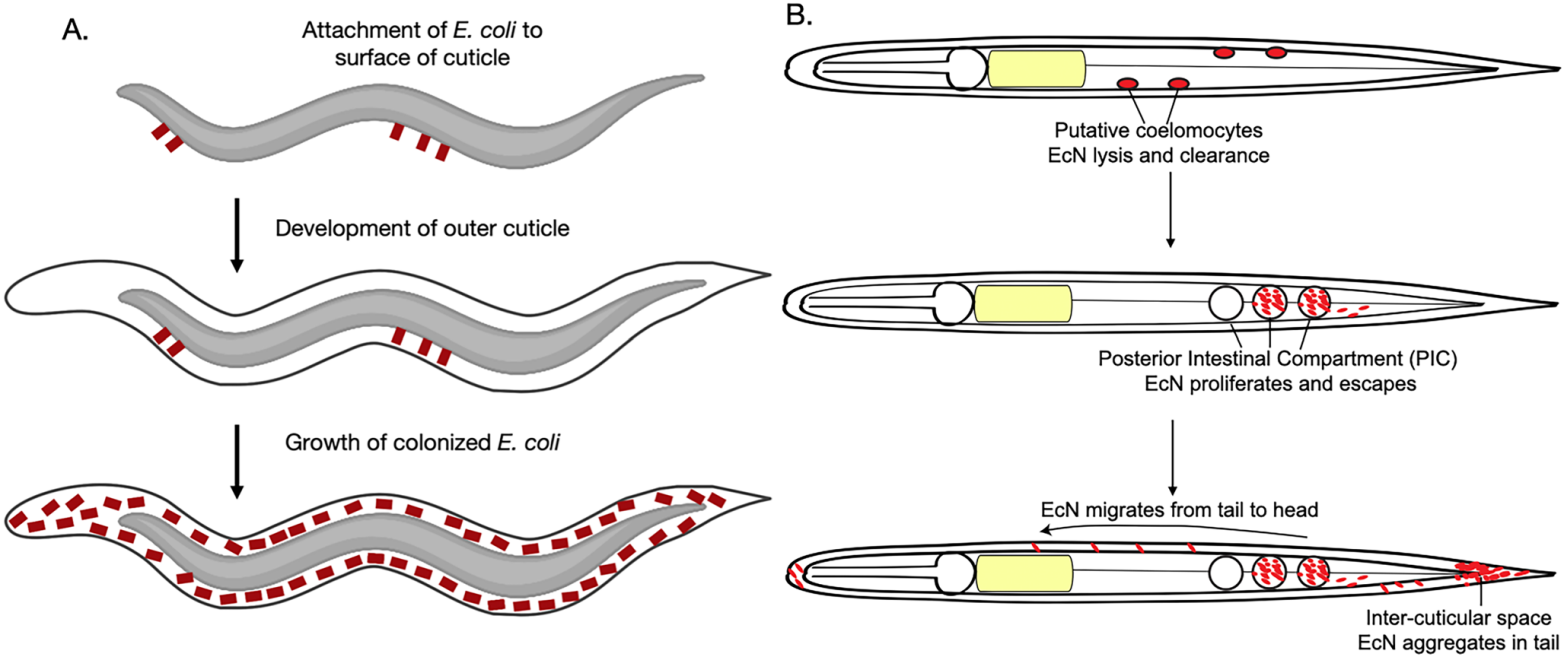
Hypothetical models of *E. coli* Nissle interaction in *S. hermaphroditum*. (A) A hypothetical model based on a previous publication of *S. scapterisci* cuticular microbes (Bonifassi et al. 1999): environmental microbes attaching to the inner cuticle during IJ formation. (B) Current hypothetical model based on data from this research: *E. coli* Nissle possibly causes mild infection in *S. hermaphroditum*.

In previous studies, environmental microbes were found in *S. scapterisci* in the ‘inter‐cuticular space’, defined as the space in between the inner cuticle of J2 and J3 larvae (Bonifassi et al. 1999). Those rod‐shaped bacteria, reported as ‘contaminants’, were thought to be wrapped into the J3 cuticle during nematode growth and moulting (Figure 7A). Note that in this research, EcN cells are temporarily localized to the space in between the collagenous inner cuticle and the outer cuticle. Based on our observations, we propose that the bacteria enter the inter‐cuticular space after being expelled from the intestine (Figure 4A,B). Within the inter‐cuticular space, EcN cells were observed to migrate from the posterior to the anterior side where they form aggregates, suggesting that these bacterial cells are indeed living. What causes the lysis of EcN cells in the cuticle is unknown, some possibilities include host nematode immune response, or an exhaustion of nutrients in the inter‐cuticular space. We attempted to colonize *S. hermaphroditum* with *Pseudomonas* species that have been reported, based on sequencing, to be part of the nematode's natural pathobiome [ref]; however, these strains were too virulent and caused rapid nematode lethality, precluding further analysis to show experimental evidence that this colonization process could be broadly applicable to interactions with other microbial species.

Despite the potential of binary symbiosis systems in environmental engineering, they remain underexplored. Engineering vertically transmitted symbionts or endosymbionts, which reside in host cell cytosol and are maternally passed through oocytes (Bright and Bulgheresi 2010), is technically challenging. In contrast, horizontally transmitted microbiomes, such as the association of *S. hermaphroditum* with *Xenorhabdus griffiniae* and EcN, are acquired extracellularly from the environment in each generation. This mode of symbiont acquisition offers key advantages for microbial bioengineering: symbiotic bacteria (including mutualistic and pathogenic) can be cultured and genetically modified ex vivo, then easily re‐associated with the host. This enables the use of engineered microbes to monitor microenvironments within host tissues or to assess external environmental conditions within relevant ecosystems. Our discovery that the easily‐engineerable bacterium EcN can reliably (albeit transiently) colonize *S. hermaphroditum* and furthermore can deliver proteins to the intercuticular space that remain protected from proteolysis over months‐long timescales in soil microcosms, opens the possibility for further research that leverages this interaction to design engineered environmental interventions based on *S. hermaphroditum*. Future research should specifically investigate whether these EcN‐expressed proteins can maintain more complex function beyond fluorescence (such as enzymatic activity) in the intercuticular space and the extent to which they are affected by, and can affect, the external environment outside of the nematode.

## 5 Conclusion

In this work, we report previously unidentified cells and tissues within *Steinernema* nematodes where EcN can localize, proliferate and lyse. We characterized sequential EcN localization within IJs, including putative immune cells (coelomocytes), posterior intestinal compartments and the inter‐cuticular space between the interior collagenous cuticle and the exterior cuticle (Figure 7B). Remarkably, mScarlet‐I proteins from EcN lysates persisted within the inter‐cuticular space for over 8 weeks under non‐sterile soil conditions. This observation challenges the paradigm that environmental microbes are passively ‘wrapped’ within moulting nematode cuticles (Figure 7A). Instead, our findings demonstrate that EcN can actively associate with soil‐dwelling *Steinernema* in a tissue‐specific manner in a niche that is separate from the nematode's natural symbiont. During EcN interaction, the immune response of *Steinernema hermaphroditum* engages the pseudocoelom, intestinal structures, and cuticle either through active invasion or passive leakage. The fact that EcN appears to activate the *S. hermaphroditum* immune response opens a new avenue of establishing *S. hermaphroditum* as a model for host–microbe interactions. The ability for engineered EcN cells to transiently persist within *Steinernema hermaphroditum*, and for heterologous proteins expressed in the EcN cells to stably persist within the nematode cuticle, opens potential avenues for enhancing *Steinernema hermaphroditum*'s potential as a biocontrol and biomonitoring system in agricultural settings.

## Author Contributions

M.C. and J.P.M. oversaw and conceptualized the project. V.C., J.P.M. and M.C. acquired preliminary data on *E. coli* colonization in *Steinernema* nematode cuticle that led to the initiation of the project. V.C., J.P.M. and M.C. designed the experiments. V.C. and M.C. performed experiments, analysed the data and wrote the manuscript. J.P.M. and R.M.M. edited the manuscript. M.C. and R.M.M. supervised the project. R.M.M., J.P.M. and M.C. acquired funding.

## Funding

M.C., V.C. and J.P.M. were supported in part by the Resnick Sustainability Institute. M.C. was also supported by Carnegie Institution for Science. J.P.M. and R.M.M. were also supported in part by the Institute for Collaborative Biotechnologies through contract W911NF‐19‐D‐0001 from the U.S. Army Research Office. The content of the information on this page does not necessarily reflect the position or the policy of the Government, and no official endorsement should be inferred.

## Ethics Statement

Experiments involving animals were conducted in accordance with institutional and national guidelines under approved protocols.

## Conflicts of Interest

The authors declare no conflicts of interest.

## Supporting information


**Figure S1:** Cell or organelle debris‐like substances in the posterior intestinal compartments (PIC) in a representative infective juvenile. Black arrows: gut granules; white arrows: cell or organelle debris‐like substances in the PIC. Scale bar = 20 μm.

**Figure S2:** A representative *S. hermaphroditum* juvenile fed on *E. coli* Nissle. mScarlet‐I expressing EcN cells are engulfed and digested in the intestinal lumen of J2 stage of *S. hermaphroditum*. ‘P’ denotes posterior end of the nematode; ‘int. lumen’ denotes ‘intestinal lumen’; ‘b.b’ denotes ‘basal bulb’; ‘AIC’ denotes ‘anterior intestinal caecum’; ‘PIV’ denotes ‘pharyngeal intestinal valve’.

**Figure S3:** A profile of 29 IJs with EcN colonization in the intestinal compartments and the inter‐cuticular space. ‘P’ denotes the posterior side of the IJ.

**Figure S4:** Outer cuticle growth and bacterial protein (mScarlet‐I) colonization in *Steinernema hermaphroditum* IJs. (A) Fractions of IJs with developed outer cuticle over 9 week's time course of experiment. (B) Fraction of developed IJs with outer cuticle that has cuticular colonization of mScarlet from EcN lysate over the 9 week's time course of experiment.

**Video S1:** Bacterial lysis within the *S. hermaphroditum* putative coelomocytes. *E. coli* Nissle expressing mScarlet is localized in the coelomocytes.

**Video S2:** Intestinal cell or organelle debris‐like substances in a representative posterior intestinal compartment (PIC).

**Video S3:** A representative infective juvenile (IJ) with two empty posterior intestinal compartments (PICs) containing nematode organelle debris‐like substances, and two PIC housing EcN cells expressing mScarlet‐I.

## Data Availability

The data that supports the findings of this study are available in the Supporting Information of this article.
