# Supplementary figures and images for: Escherichia coli Nissle 1917 Occupies Previously Undocumented Host Niches in the Insect‐Parasitic Nematode Steinernema hermaphroditum
Source: Environ Microbiol Rep. 2026 Apr 5;18(2):e70326. doi: 10.1111/1758-2229.70326 (PMC13052097; doi:10.1111/1758-2229.70326)

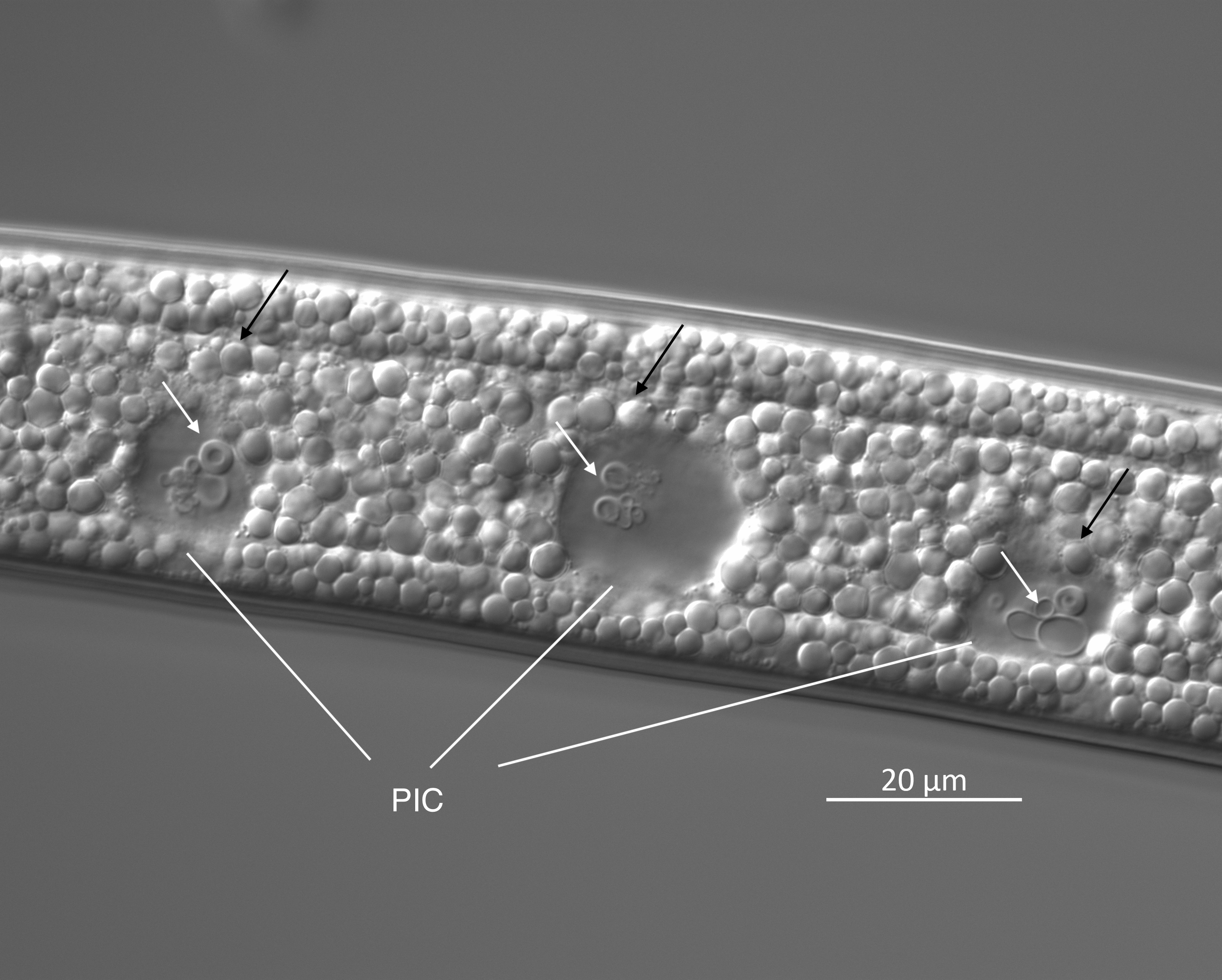

Supplement: Supplementary file 1 — Figure S1: Cell or organelle debris‐like substances in the posterior intestinal compartments (PIC) in a representative infective juvenile. Black arrows: gut granules; white arrows: cell or organelle debris‐like substances in the PIC. Scale bar = 20 μm. [file EMI4-18-e70326-s005.png]

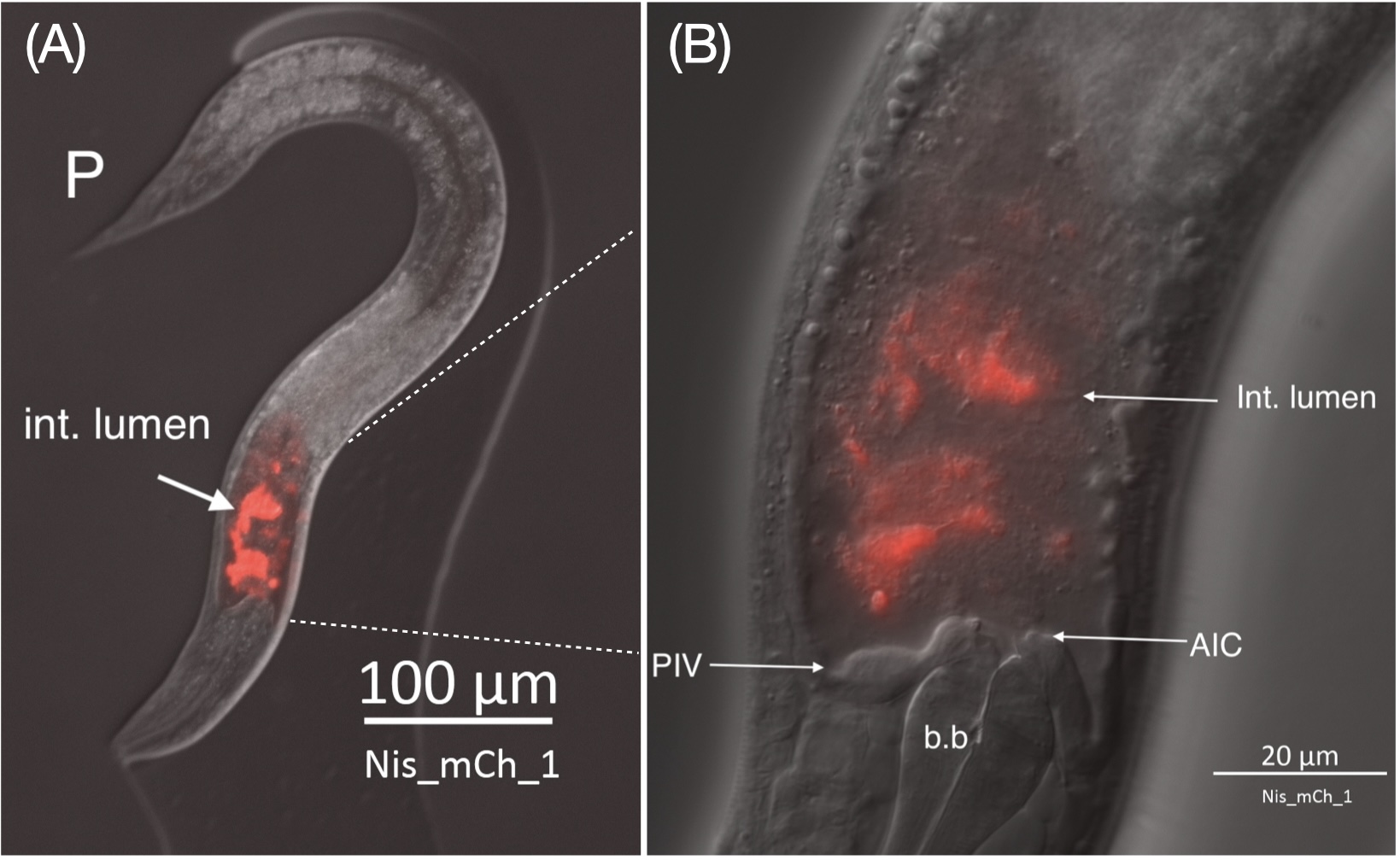

Supplement: Supplementary file 2 — Figure S2: A representative S. hermaphroditum juvenile fed on E. coli Nissle. mScarlet‐I expressing EcN cells are engulfed and digested in the intestinal lumen of J2 stage of S. hermaphroditum. ‘P’ denotes posterior end of the nematode; ‘int. lumen’ denotes ‘intestinal lumen’; ‘b.b’ denotes ‘basal bulb’; ‘AIC’ denotes ‘anterior intestinal caecum’; ‘PIV’ denotes ‘pharyngeal intestinal valve’. [file EMI4-18-e70326-s006.jpeg]

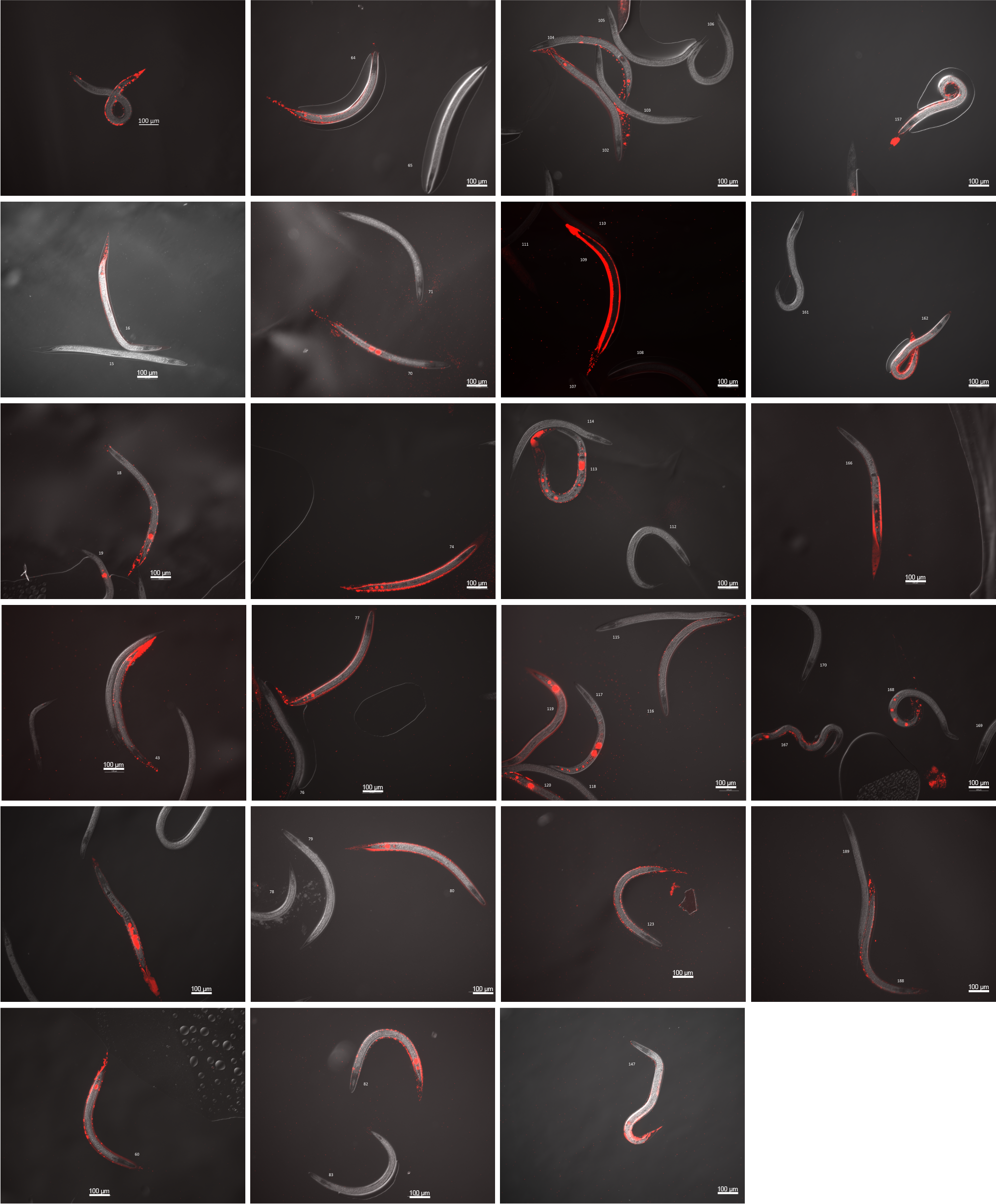

Supplement: Supplementary file 3 — Figure S3: A profile of 29 IJs with EcN colonization in the intestinal compartments and the inter‐cuticular space. ‘P’ denotes the posterior side of the IJ. [file EMI4-18-e70326-s004.png]

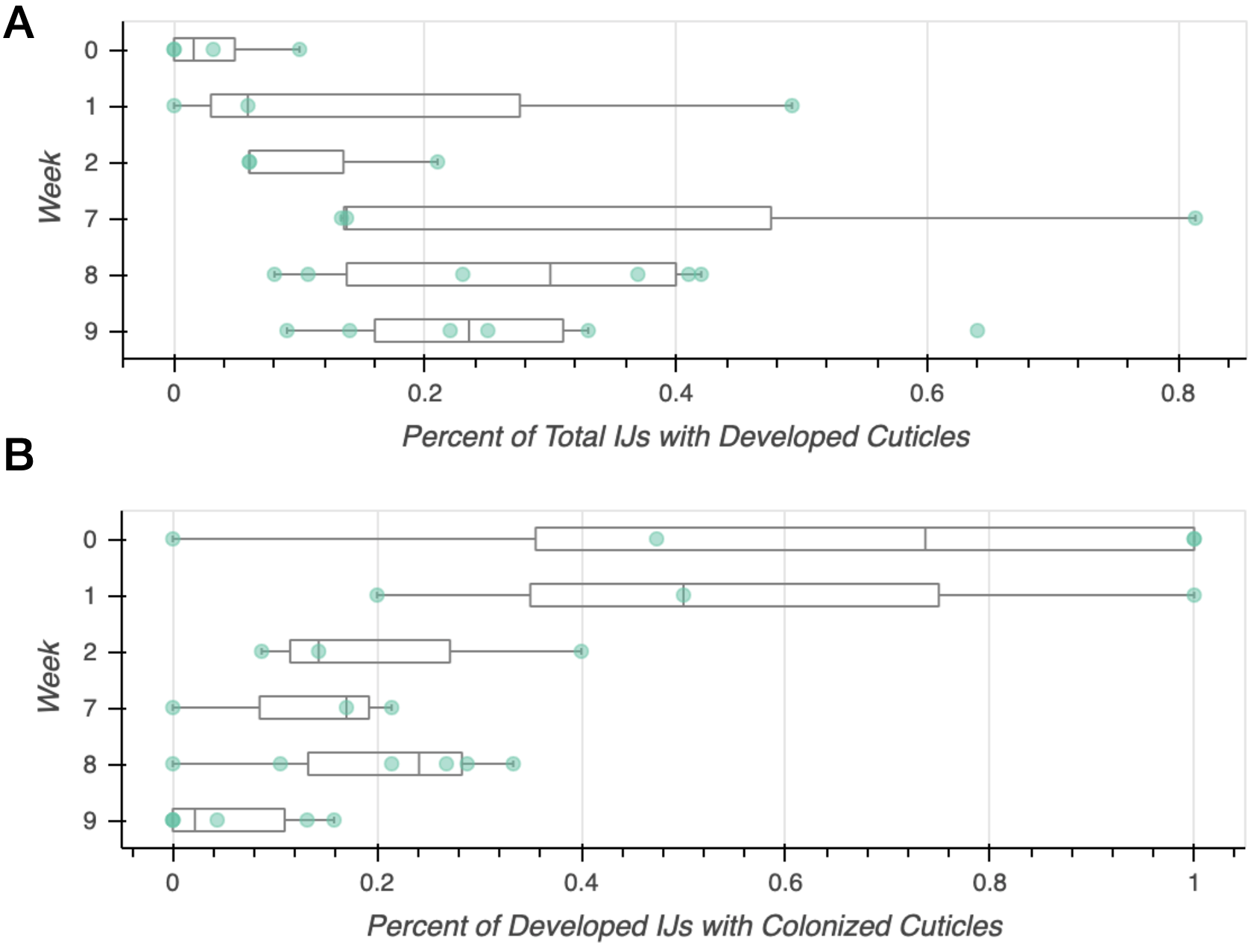

Supplement: Supplementary file 4 — Figure S4: Outer cuticle growth and bacterial protein (mScarlet‐I) colonization in Steinernema hermaphroditum IJs. (A) Fractions of IJs with developed outer cuticle over 9 week's time course of experiment. (B) Fraction of developed IJs with outer cuticle that has cuticular colonization of mScarlet from EcN lysate over the 9 week's time course of experiment. [file EMI4-18-e70326-s007.png]
